# Novel Siglec-15-Sia axis inhibitor leads to colorectal cancer cell death by targeting miR-6715b-3p and oncogenes

**DOI:** 10.3389/fimmu.2023.1254911

**Published:** 2023-10-06

**Authors:** Mohammed Saqif Ahmad, Maria Braoudaki, Hershna Patel, Irshad Ahmad, Shagufta, Shoib Sarwar Siddiqui

**Affiliations:** ^1^ School of Life and Medical Sciences, University of Hertfordshire, Hatfield, United Kingdom; ^2^ Department of Biotechnology, School of Arts and Sciences, American University of Ras Al Khaimah, Ras Al Khaimah, United Arab Emirates

**Keywords:** colorectal cancer, Siglec-15, sialic acid, inhibitor, miRNA, gene expression

## Abstract

Siglecs are well known immunotherapeutic targets in cancer. Current checkpoint inhibitors have exhibited limited efficacy, prompting a need for novel therapeutics for targets such as Siglec-15. Presently, small molecule inhibitors targeting Siglec-15 are not explored alongside characterised regulatory mechanisms involving microRNAs in CRC progression. Therefore, a small molecule inhibitor to target Siglec-15 was elucidated *in vitro* and microRNA mediated inhibitor effects were investigated. Our research findings demonstrated that the SHG-8 molecule exerted significant cytotoxicity on cell viability, migration, and colony formation, with an IC_50_ value of approximately 20µM. SHG-8 exposure induced late apoptosis *in vitro* in SW480 CRC cells. Notably, miR-6715b-3p was the most upregulated miRNA in high-throughput sequencing, which was also validated via RT-qPCR. MiR-6715b-3p may regulate *PTTG1IP*, a potential oncogene which was validated via RT-qPCR and *in silico* analysis. Additionally, molecular docking studies revealed SHG-8 interactions with the Siglec-15 binding pocket with the binding affinity of -5.4 kcal/mol, highlighting its role as a small molecule inhibitor. Importantly, Siglec-15 and PD-L1 are expressed on mutually exclusive cancer cell populations, suggesting the potential for combination therapies with PD-L1 antagonists.

## 1 Background

Colorectal cancer (CRC) has been referred to as the fourth most diagnosed cancer worldwide and the third leading cause of patient mortality in humans (1). Approximately, 1,931,590 new cases of CRC were reported with 935,137 patient deaths worldwide in 2020 (1). CRC has become increasingly prevalent worldwide as well, with statistical trends indicated cancer-related deaths could reach as high as 71.5% by 2035 (2). Currently, conventional treatments for CRC patients have fallen short as successful therapeutic strategies, due to lacking in patient response, severe side effects and modest specificity consequently resulting in patient mortality and/or tumour recurrence (3). The recent trend of immunotherapy in solid cancers such as melanoma have shown success with blocking antibodies including Nivolumab and Pembrolizumab (4).

The blocking antibodies have also been approved for the treatment of CRC that have a high number of mutations with high microsatellite instability type (MSI-H) tumours (5). However, immunotherapy treatment has some limitations such as effectiveness in limited patient numbers, with acquired resistance to treatment a likely possibility (3). Therefore, a necessity to develop a novel approach to proficiently treat CRC has emerged.

Sialic acid-binding immunoglobulin-type of lectin 15 (Siglec-15) is a recently emerging immune checkpoint protein involved in suppressing the immune system and inducing cancer progression (6). An overexpression of Siglec-15 is commonly observed in various cell types. Most notably, it is upregulated on the surface of tumour cells. Pan-cancer analysis identified that this included various cancers such as CRC, non-small cell lung cancer (NSCLC), lung squamous cell carcinoma (SCC), ovarian cancer (OV), and others (7). Additionally, high Siglec-15 expression has been shown to correlate with increased MSI-H type tumours of CRC patients (8). Interestingly, the expression of both PDL-1 and Siglec-15 are mutually exclusive on cancer cell populations. Therefore, further treatments targeting Siglec-15 are required for Siglec-15 positive tumours.

MicroRNAs (miRNAs) are small non-coding RNA molecules approximately 20-25 nucleotides long capable of binding to the 3’ untranslated region (UTR) of mRNA target molecules and regulating gene expression post-transcriptionally (9). Several miRNAs have shown differential expression profiles in CRC malignancies indicating oncogenic/tumour suppressive roles within CRC development and progression (10). The identification of specific miRNAs that is explicitly involved in CRC progression remains largely unclear. Interestingly, this study has given insights into the possible interactions between miRNAs and oncogenes for considerable strategies for the treatment of *SIGLEC15* positive CRC tumours.

The class of compounds, β-amino ketones, bearing an amino group at the beta carbon to ketone functional group is an important pharmacophoric feature employed in the synthesis of numerous natural products, drugs, and bioactive molecules (11). These compounds are biologically active scaffolds that exhibit a wide range of activities including anticancer, anti-inflammatory, antibacterial, antiviral, and antidiabetic etc. (12). In addition, several β-amino derived drugs are currently available for the treatment of various diseases and disorders (13).

Small drug-like molecules that modulate the immune system by targeting defined pathways or cells can improve the effectiveness of cancer immunotherapy (14). As far as our understanding with the literature, small molecules have not been explored for the treatment of Siglec-15 positive tumours by targeting Siglec-15. Considering the β-amino ketone’s biological significance, we synthesised 3-(4-bromophenyl)-1-phenyl-3-(phenylamino)propan-1-one (SHG-8) through a one-step catalytic route using an environmentally benign solid acid nano-catalyst. We believed that the molecule SHG-8 being a small molecule in nature could possibly interact with the therapeutic target Siglec-15 and this could be our leading point towards the development of strategies for CRC treatment. Furthermore, the β-amino ketones affecting miRNA expression and subsequent interactions with genes and pathways in CRC development remains largely unknown. Therefore, it may be possible to identify miRNAs as targets for therapeutic treatment by elucidating their role in CRC development and their association to *SIGLEC15* regulation.

## 2 Materials and methods

### 2.1 Reagents and instruments

All the starting materials, reagents, and solvents were obtained from Merck and Spectrochem. The synthesis and characterization data of the sulfonic acid-functionalized silica nanospheres (SAFSNS), an environmentally benign solid acid nano-catalyst is available in our recent publication (15). The progress of the synthesis reaction of the SHG-8 molecule was monitored by using thin-layer chromatography (TLC) on silica gel plates and the spots were visualized under ultraviolet light (UV, λ254 nm). Infrared spectra were recorded on a PerkinElmer FTIR spectrophotometer. Mass spectra were measured with an electrospray (ESI-MS) on Shimadzu LCMS-spectrophotometer. ^1^H and ^13^C NMR spectra of the SHG-8 molecule were recorded using the Bruker AV-400 NMR spectrometer with tetramethylsilane as an internal standard. High Resolution Mass Spectrum (HRMS) was recorded on an Agilent 6540 HD Accurate Mass QTOF/LC/MS with electrospray ionization (ESI) technique.

#### 2.1.1 Synthesis of 3-(4-bromophenyl)-1-phenyl-3-(phenylamino)propan-1-one

The SHG-8 molecule was prepared in a one-step catalytic reaction by a stirring mixture of acetophenone (1.1 mmol), 4-Bromo benzaldehyde (1 mmol), and aniline (1 mmol) in 1mL of ethanol at room temperature (RT), the sulfonic acid functionalized silica nano spheres (SAFSNS) nano-catalyst (0.03 g) was added. The reaction mixture was stirred at RT for 4h. After completion of the reaction as indicated by Thin Layer Chromatography (TLC), the reaction mixture was placed at ambient temperature to evaporate ethanol and water to obtain a yellow solid. The solid was dissolved in dichloromethane (5mL) at 35°C and filtered to remove the catalyst. The crude product was purified by the recrystallization technique, using an ethanol solvent to afford compound SHG-8 as a light-yellow solid: yield 85%.

### 2.2 Cell culture and treatments

Human CRC SW480 and GBM cell line U87MG were obtained from ATCC (ATCC, Virginia, USA). Human monocytic cell lines THP-1 and U937 were obtained from ATCC (ATCC, Virginia, USA). Additionally, the human normal colon tissue HcoEPiC cell line was obtained from iXcells Biotechnologies (iXcells Biotechnologies, San Diego, USA). The adherent cell lines SW480 and U87MG were grown using DMEM (Gibco, Bleiswijk, Netherlands) and MEM (Gibco, Bleiswijk, Netherlands) media respectively, and supplemented with 10% FBS (Gibco, Bleiswijk, Netherlands) and 1% penicillin/streptomycin (Gibco, Bleiswijk, Netherlands). Both THP-1 and U937 suspension cell lines were grown using DMEM (Gibco, Bleiswijk, Netherlands) media respectively, and supplemented with 10% FBS (Gibco, Bleiswijk, Netherlands) and 1% penicillin/streptomycin (Gibco, Bleiswijk, Netherlands). Both cell lines were also differentiated into macrophages 48h post- phorbol 12-myristate 13-acetate (PMA) treatment (50ng/mL) for determining cytokine expression. The HcoEPiC cell line was grown using epithelial cell growth media (iXcells Biotechnologies, San Diego, USA) supplemented with 1% antibiotic-antimycotics (iXcells Biotechnologies, San Diego, USA). All cell lines were incubated at 37°C, 5% CO_2_ and further experimental work was conducted upon reaching a minimum confluency of 80%. SHG-8 was solubilised in DMSO at a stock concentration of 10mM and further diluted for various assay experiments.

### 2.3 MTT assay

SW480 CRC cells (1.5x10^4^ cells/well) and HcoEPiC cells were seeded at a cellular density of 2.5x10^4^ cells/well onto a 96 well-plate and left to incubate at 37°C, 5% CO_2_ overnight. Following cell adherence, all wells were treated with varying SHG-8 concentrations: 20µM, 40µM, 60µM, 80µM, 100µM and were incubated for 24h at 37°C, 5% CO_2_. Following overnight incubation, all wells were treated with 20µL MTT (0.5mg/mL) and were left to incubate for a further 2h at 37°C. The MTT was then removed, and the remaining crystals were treated and solubilised with isopropanol for 30 min on a shaker. Absorbance was then recorded at 540nm using a CLARIOstar plus multi-mode microplate reader (BMG LABTECH, Aylesbury, UK). Average percentage cell viability was calculated.

### 2.4 Migration assay

In total, 4x10^5^ cells/well were seeded onto a 12-well plate and left to incubate overnight at 37°C, 5% CO_2_. Following cell adherence, vertical and horizontal scratches were made for each well using a 2µL pipette tip. The wells were then washed with PBS twice. All wells were treated with SHG-8 at 10µM and 40µM concentrations alongside control treatments. Images of the wound area were taken at 0h, 24h, 48h using an Olympus CKX41 inverted microscope (Olympus Life Science Solutions, Stansted, UK) at 4x magnification. All images taken relating to the wound area were then analysed using ImageJ analysis. Plugins for wound healing analysis were obtained from a previous study (16).

### 2.5 Colony formation assay

In total, 1x10^3^ cells/well were seeded onto a 12-well plate and left to incubate overnight at 37°C, 5% CO_2_. Following cell adherence, the cells were then treated in triplicate with SHG-8 concentrations at 10µM and 40µM and were placed into the incubator for 24h. Following the 24h treatment period, the treatment was replaced with fresh media and left to incubate at 37°C, 5% CO_2_ for a period of 7 days. The incubation period was determined by the total number of colonies formed in the DMSO control (colonies were determined as a cluster of cells totalling larger >30 cells). Following the 7-day incubation period, all wells were fixed with 4% PFA for total of 20 min. After fixation, all wells were washed with PBS three times for 5 min each before being stained with 0.1% crystal violet (Pro-Lab Diagnostics, Wirral, UK) for 45 min on a rocker. Following staining, all wells were washed with PBS for 3 min and images of all colonies were taken at 4x and 10x magnifications using an Olympus CKX41 inverted microscope (Olympus Life Science Solutions, Stansted, UK).

### 2.6 Nuclear fragmentation assay

SW480 cells (4x10^5^ cells/well) were seeded onto coverslips within a 12 well-plate and left to adhere overnight at 37°C, 5% CO_2_. The cells were treated with: DMSO, 10µM and 40µM SHG-8 as well as 100µM cisplatin for 24h. After the incubation period, all wells were washed with PBS. The cells were then fixed with 4% PFA for a period of 15-20 minutes and kept at RT. The fixation was removed and washed again with PBS. The coverslips were removed and placed onto slides with DAPI Antifade Mounting Medium (2Bscientific, Hatfield, UK). Fluorescent images were taken with an EVOS XL Core Imaging system (Fisher Scientific, Hemel Hempstead, UK) at 40x magnification with the DAPI filter.

### 2.7 Annexin-V/propidium iodide staining

To determine the stage of apoptosis induction post SHG-8 treatment, a total of 3x10^6^ cells/well were seeded onto a 6 well plate and left to adhere overnight. Following cellular adherence, all wells were treated with control conditions and SHG-8 at 10μM and 40μM conditions for a period of 24h. Following treatment, all wells were washed with ice-cold PBS and 1x10^6^ cells were harvested to 100μL suspensions. The SW480 cells were treated with 1μL of RNase A (10mg/mL) before being labelled with Annexin-V FITC (5μL) and propidium iodide (100μg/mL). Using the dead cell apoptosis kit with Annexin-V FITC and propidium iodide for flow cytometry (Invitrogen, Inchinnan, UK) to each 100μL cell suspension following the manufacturer’s instructions. The cell suspensions were left to incubate at RT for a period of 15 min. Following incubation, 400μL of 1x binding buffer was added to each sample and left on ice before analysing via the Guavasoft 3.1.1 software and guava easyCyte HT system (Merck Millipore, Watford, UK).

### 2.8 Cell cycle arrest via flow cytometry method

Cell cycle analysis of SW480 cells at sub-G0/G1/S/G2/M phases post SHG-8 treatment was performed via PI staining using the dead cell apoptosis kit with Annexin-V FITC and propidium iodide (Invitrogen, Inchinnan, UK) as per the manufacturer’s instructions. The cells were harvested and treated with 100µg/mL PI and left to incubate for 15 min at RT. The cell suspensions were analysed using the Guavasoft 3.1.1 software and guava easyCyte HT system (Merck Millipore, Watford, UK).

### 2.9 ROS assay

To determine ROS production, SW480 (1.5x10^4^ cells/well) cells were seeded onto a 96 well-plate and left to incubate overnight at 37°C, 5% CO_2_. ROS production was determined using the Reactive Oxygen Species (ROS) Detection Assay Kit (Abcam, Cambridge, UK). Following cell adherence, 20µL ROS red dye was added to each of the wells and left to incubate at 37°C, 5% CO_2_ for 1 hour. After the incubation period, 20µL of each sample treatment was added to each of the wells following manufacturer’s instructions. Fluorescence readings were obtained at 15-, 30-, 45-, 60- and 75-minute intervals at Ex/Em = 520/605 nm using a CLARIOstar plus multi-mode microplate reader (BMG LABTECH, Aylesbury, UK).

### 2.10 Molecular docking study methods

A model of the Siglec-15 structure was downloaded from the AlphaFold Protein Structure Database with the corresponding UniProt entry Q6ZMC9, as there was no experimental structure available in the Protein Data Bank (17). The COACH server was used to predict protein-ligand binding sites on the AlphaFold model (18). The chemical structure of SHG-8 was obtained in SDF format. Molecular docking was carried out using the web app Webina 1.0.3 which runs Autodock Vina entirely in the web browser (19). The Siglec-15 AlphaFold model was uploaded as the receptor and the SHG-8 SDF file was uploaded as the ligand, both files were automatically converted to the PDBQT format for docking. The grid box was centred around the ARG143 binding site, and the parameters were set as follows: centre_x = -23, center_y = 6, center_z = 13.272 and size_x = 28, size_y =21, size_z = 22. The default settings of 2 CPUs and exhaustiveness of the global search set as 8 were used for docking.

### 2.11 IF staining

SW480 and U87MG cells (3x10^5^ cells/well) were seeded onto coverslips onto a 12-well plate and left to incubate overnight. Following cell adherence, all wells were washed with PBS three times for 5 min each and then fixed with 4% PFA for a total of 15 min. With the fixation removed and washed with PBS, all wells were treated with a 1% BSA/PBS blocking buffer for 1h before incubating the primary Siglec-15 antibody (Life Technologies Limited, Renfrewshire, UK) at a dilution of 1:500 at 4°C overnight. Following this, the antibody is removed and washed with PBS before treating with an anti-rabbit Alexa-488 secondary antibody (Life Technologies Limited, Renfrewshire, UK) at a dilution of 1:500 for 1h at RT. The coverslips were then placed onto microscope slides and visualised with an EVOS XL Core Imaging system (Fisher Scientific, Hemel Hempstead, UK) at 40x magnification with the DAPI and GFP filter.

### 2.12 Siglec-15 expression via flow cytometry method

SW480 cells were seeded at a density of 3x10^6^ cells/well onto a 6 well plate and left to adhere overnight. Following cell adherence, the cells were treated with varying SHG-8 concentrations and controls for 24h. Post-treatment, the cells were washed with ice-cold PBS and harvested with 10mM EDTA at a cellular density of 1x10^6^ cells and resuspended in 2% BSA : PBS (fluorescence activated cell sorting buffer- FACS buffer). The cells were washed with ice-cold PBS and centrifuged at 200xg for 5 min twice. The cell samples were then resuspended in FACS buffer alongside the addition of a monoclonal Siglec-15 primary antibody (R&D Systems, Minneapolis, USA) (0.25µg) raised in mouse and left to incubate on ice for a period of 45 min. Following incubation, the cells were washed with ice-cold PBS and centrifuged at 200xg for 5 min twice before being resuspended in FACS buffer. Each cell suspension was treated with an anti-mouse Alexa- 488 secondary antibody (Life Technologies Limited, Renfrewshire, UK) and left to incubate on ice for a period of 30 min in the dark. After the secondary antibody incubation, the cells were washed once more with ice-cold PBS and centrifuged before being resuspended in FACS buffer for analysis via the guava easyCyte HT system (Merck Millipore, Watford, UK) and Guavasoft 3.1.1 software.

### 2.13 Cytokine expression via enzyme-linked immunosorbent assay method

The secretion of pro-inflammatory cytokines TNF-α and IL-1β were evaluated in THP-1 and U937 differentiated macrophages following SHG-8 treatment. Both cell lines were differentiated into macrophages with PMA (50ng/mL) for 48h prior to 40µM SHG-8 treatment and LPS (50ng/mL) stimulation for 24h. The supernatant was harvested and were placed as duplicates onto microwell strips alongside standards using the respective cytokine human ELISA kit (Life Technologies Limited, Renfrewshire, UK) after several wash steps as per the manufacturer’s instructions. 50µL of biotin-conjugate were added to all the strips and were left to incubate at RT for 2h on a microplate shaker. Following the incubation period, the strips were washed and 100µL of streptavidin-HRP were added to all the strips for 1h. The strips were then washed again following the incubation period. After consecutive wash steps, 100µL of TMB substrate solution were added to all wells and left to incubate at RT for 10 min or until a noticeable colour change was observed. Following colour development, 100µL of stop solution were added to each of the strips and was then measured at 620nm using a CLARIOstar microplate reader (BMG LABTECH, Aylesbury, UK).

### 2.14 RNA extraction and RT-qPCR methods

Total RNA was extracted from SW480 cultured cells 24hrs post-treatment under the following conditions: DMSO, SHG-8 10μM and 40μM as well as 100μM cisplatin using the TRIzol method (Life Technologies Limited, Renfrewshire, UK). RNA quality and quantity of all treated samples for gene and miRNA expression analyses were evaluated using a Nanodrop ND-1000 spectrophotometer UV-Vis Nanogen Inc. (Marshall Scientific, Hampton, USA). Following quantification, expression measurement analysis of treated samples had undergone Dnase treatment with the Rnase-free Dnase set respectively (Qiagen, Hilden, Germany). In brief, the mRNA samples for gene analysis were directly subjected to cDNA synthesis and subsequent RT-qPCR was performed using the High-Capacity cDNA reverse transcription kit (Applied Biosystems, Massachusetts, USA) followed by the Taqman Fast Advanced Mastermix, no UNG (Applied Biosystems, Massachusetts, USA) and PowerUp SYBR Green Mastermix (Applied Biosystems, Massachusetts, USA for probe-based and SYBR green qPCR analyses. SYBR green primer sequences involved in analysis are as follows: *GAPDH* forward (GGAGCGAGATCCCTCCAAAAT) and reverse (GGCTGTTGTCATACTTCTCATGG) and *PTTG1IP* forward (GTCTGGACTACCCAGTTACAAGC) and reverse (CGCCTCAAAGTTCACCCAA). All treated samples collected for miRNA expression analysis were further purified and subjected to miRNA enrichment with the miRvana miRNA Isolation Kit (Life Technologies Limited, Renfrewshire, UK). Following this, miRNA samples were further quantified for their RNA quality and quantity prior to reverse transcription with the TaqMan MicroRNA Reverse Transcription kit (Applied Biosystems, Massachusetts, USA). RT-qPCR of individual Taqman miRNA assays (Applied Biosystems, Massachusetts, USA) were performed using the TaqMan Universal Mastermix II, no UNG (Applied Biosystems, Massachusetts, USA) following manufacturer’s instructions. All samples were performed in triplicate using the QuantStudio 7 Flex Real-Time PCR System (Applied Biosystems, Massachusetts, USA). GAPDH and U47 used as housekeeping controls respectively. Relative expression of *SIGLEC15* and candidate miRNAs and genes were performed using the **2^–ΔΔCt^ method.**

### 2.15 Small-RNA sequencing

To determine the differential expression of miRNAs treated with SHG-8, sRNA-seq was outsourced to Biomarker Technologies (Biomarker Technologies, Inc., CA, USA). SW480 cells were subjected to 24hrs SHG-8 treatment prior to total RNA extraction via TRIzol reagent method. The purity and quantity of each RNA sample was processed using a Nanodrop ND-1000 spectrophotometer UV-Vis Nanogen Inc. (Marshall Scientific, Hampton, USA) and 4150 TapeStation System (Agilent Technologies, California, USA). All samples were recorded with RIN numbers >7.0. The resulting data was subjected to Kyoto Encyclopedia of Genes and Genomes (KEGG), Gene Set Enrichment Analysis (GSEA) and Gene Ontology (GO) to identify enriched pathways, biological processes, molecular function, and cellular components in relation to CRC.

### 2.16 UALCAN datamining

The UALCAN database (http://ualcan.path.uab.edu, accessed on 9 May 2023) was utilised for TCGA cancer genomic analysis and is presented as a tool for cancer transcriptomics. Utilising TCGA genomic data, the expression of selected miRNAs was compared among both tumour and normal subgroups. *PTTG1IP* expression in normal and tumour subgroups were also compared at the protein level.

### 2.17 Statistical analysis

MTT assays for both SW480 and HcoEPiC cell lines and colony formation assays evaluating the effect of SHG-8 on cancer cells and cytokine secretion of SHG-8 treated THP-1 and U937 differentiated macrophages were compared using the one-way ANOVA statistical test followed by Dunnett’s multiple comparison *post-hoc* test to identify any statistical significance. The migration assay compared all treatment conditions with Welch’s one-way ANOVA followed by Dunnett’s multiple comparison *post-hoc* test. Additionally, determining the effect of ROS production in the induction of apoptosis used the two-way ANOVA statistical test followed by Dunnett’s multiple comparison *post-hoc* test. RT-qPCR expression analyses of *SIGLEC15*, gene targets and candidate miRNAs were compared with the one-way ANOVA statistical test and Dunnett’s multiple comparison test. Genomic and proteomic data of candidate miRNAs and gene targets between normal and tumour subgroups were compared with an unpaired student’s t-test. Statistical analysis was performed using GraphPad prism 8. *P<0.05, **P<0.01, ***P<0.001, **** P<0.0001 were considered as statistically significant.

## 3 Results

### 3.1 Synthesis and characterisation of β-amino ketones

**Figure 1:**
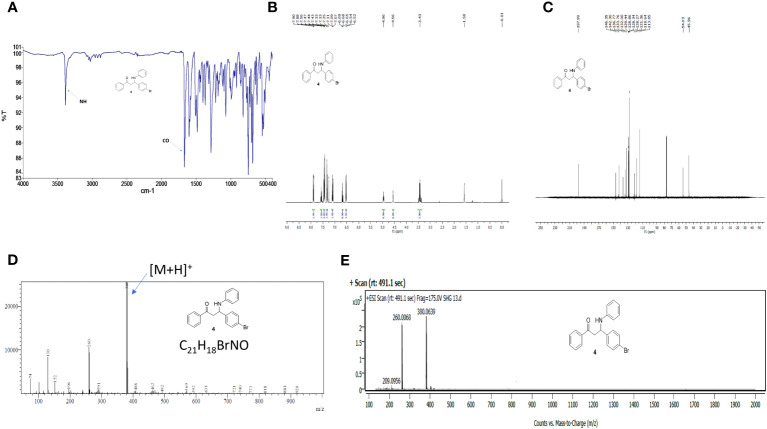
Spectra of compound 4 (SHG-8). **(A)** FTIR spectrum. **(B)** ^1^H NMR spectrum in CDCl_3_. **(C)** ^13^C NMR spectrum CDCl_3._ **(D)** ESI-MS spectrum. **(E)** High-resolution mass spectrum (HRMS).

The 3-(4-bromophenyl)-1-phenyl-3-(phenylamino)propan-1-one SHG-8 molecule was synthesised efficiently using our in-house developed environmentally benign solid acid nano-catalyst (SAFSNS) at ambient temperature and in good yield. The compound SHG-8 was characterized by spectroscopic techniques including IR, Mass, ^1^HNMR and ^13^C NMR and melting point that accurately matches with the literature data (20). The observed melting point of compound SHG-8 was 122-126°C that corresponds to the literature melting point (123–127°C). The sharp peak at 3391 (medium) and 1667 (strong) cm^-1^ in IR spectrum indicated the presence of -NH and C=O group respectively (**Figure 1A**). In the ^1^H NMR spectrum of compound SHG-8 (**Figure 1B**), the two COCH_2_ protons appeared as multiplet at δ 3.41-3.45 and the one NH proton appeared as singlet at δ 4.56. The one CH proton next to NH group appeared as a triplet at δ 4.96. The fourteen aromatic protons were observed in the downfield region, including a doublet at δ 6.53 (*J* = 8 Hz) for two protons, triplet at δ 6.68 for one proton, multiplet at δ 7.07-7.11 for two protons, doublet at δ 7.31 (*J* = 8Hz) for two protons, multiplet at δ 7.42-7.57 for four protons, multiplet at δ 7.55-7.57 for one proton and a doublet at δ 7.79 (*J* = 8 Hz) for two protons. The ^13^C NMR spectrum of compound SHG-8 (**Figure 1C**) was also in full agreement with the assigned structures showing fifteen peaks at δ 45.9, 54.1, 113.8, 118.6, 121.3, 128.2, 128.3, 128.8, 129.4, 132.0, 133.7, 136.7, 142.3, 146.3, 197.9. The MS (ESI) spectra showed M++H peak at m/z 380 (**Figure 1D**) and in the HRMS (ESI) spectra (**Figure 1E**) the m/z peak is found at 380.0639 matching with the calculated m/z for C_21_H_19_BrNO [M+H]^+^ which is 380.0645, showing the delta value 0.0006.

### 3.2 Cytotoxic ability of SHG-8 on CRC cells

**Figure 2:**
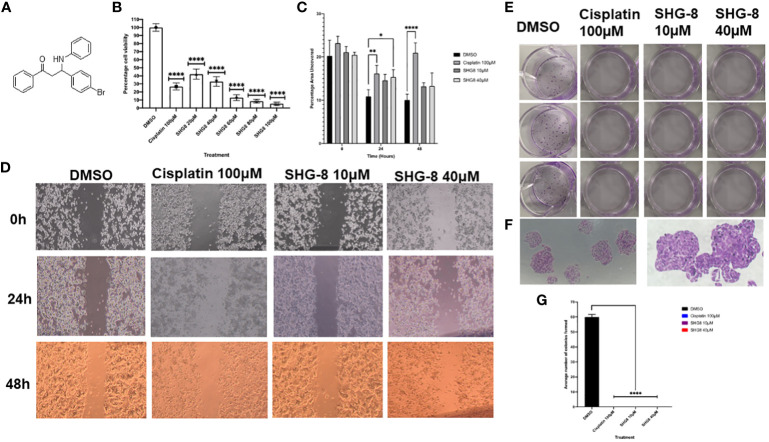
The anti-tumorigenic effect of the β-amino ketone SHG-8 against SW480 CRC cancer cells *in vitro*. **(A)** The molecular structure of compound SHG-8. **(B)** MTT assay was conducted to determine the IC_50_ of SHG-8 24 h post-treatment and to evaluate its cytotoxic effects *in vitro* (N=5). Data is presented as mean ± SEM. One-Way ANOVA statistical test followed by Dunnett’s multiple comparison *post-hoc* test **** P<0.0001. **(C)** Migration assay was conducted to evaluate the effect of SHG-8 on cellular migration following 48h post-treatment (N=3). Data is presented as mean ± SEM. Welch’s One-Way ANOVA followed by Dunnett’s multiple comparison *post-hoc* test. *P<0.05, **P<0.01, ***P<0.001, **** P<0.0001. **(D)** 4x microscopic images taken of the wound area across all treatment conditions over the 48h treatment period. Images were analysed via ImageJ with inserted plugins outlined by Suarez-Arnedo et al., 2020. **(E)** Visual image outlining the 12-well plate utilised for the colony formation assay 7-days post-treatment. **(F)** 4x and 10x microscopic images taken of SW480 cellular colonies of the DMSO condition 7-days post-treatment. Colonies were counted as clusters of cells >30. **(G)** Colony formation assay outlining the effect of SHG-8 on SW480 colony formation and cell proliferation 7 days post-treatment (N=3). Data is presented as mean ± SD. One-way ANOVA statistical test followed by Dunnett’s multiple comparison *post-hoc* test. **** P<0.0001 were considered as statistically significant.

The cytotoxic effect of SHG-8 was evaluated *in vitro* on the SW480 CRC cell line via MTT, wound healing and colony formation assays (**Figure 2**). Using cisplatin as a positive control, SHG-8 has demonstrated a significant dose-dependent cytotoxic effect on the SW480 cell line with an IC_50_ value of ~20μM, and significant cell death of ~90% upon reaching 80-100μM concentrations (*p<0.0001*) (**Figure 2B**). Wound healing has also shown an anti-tumour effect on cellular migration with the SHG-8 40μM concentration showing significant reduction of 15.37% at 24 hours (*p<0.05*) (**Figure 2C**). Additionally, both SHG-8 10µM and 40µM concentrations have shown similar reductions for 48 hours at 13.22% and 13.28% respectively in comparison to the DMSO control, possibly only effective in the early time points. SHG-8 has also shown a significant negative effect on colonisation of cancer cells (**Figures 2E-G**). Both 10µM and 40µM SHG-8 concentrations produced no colonies on the plate as also observed with the cisplatin control (*p<0.0001*) (**Figure 2G**).

### 3.3 SHG-8 induced apoptosis in SW480

**Figure 3:**
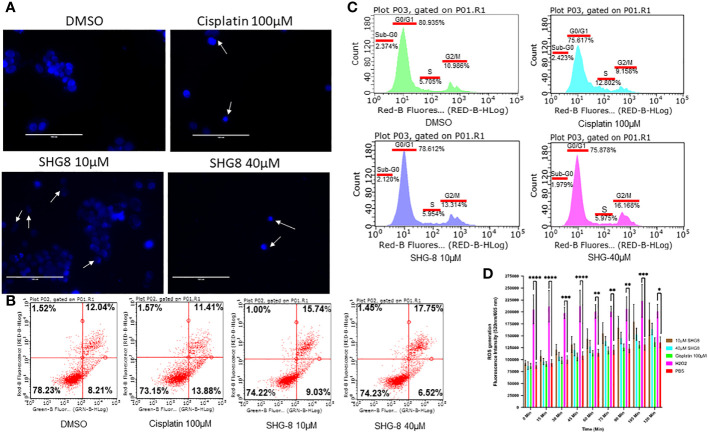
SHG-8 induced concentration-dependant apoptosis however the mechanism of action is not ROS-mediated. **(A)** Nuclear fragmentation assay determined if SHG-8 could induce apoptosis at various treatment concentrations, white arrows indicate nuclear fragments and irregular cellular morphology 24 h post-treatment at all treated conditions (N=3). **(B)** Annexin-V/propidium iodide (PI) staining of SW480 cells treated with SHG-8 at 10µM and 40µM conditions via flow cytometry to determine apoptosis induction post-treatment, dot plots are divided into four quadrants. FITC negative and PI positive (upper left quadrant) represents cells undergoing necrosis. FITC positive and PI positive (upper right quadrant) represents cells undergoing late apoptosis. FITC negative and PI negative (lower left quadrant) represents live viable cells. FITC positive and PI negative (lower right quadrant) represents cells undergoing early apoptosis. **(C)** Cell cycle arrest analysis of SW480 cells at sub-G0/G1/S/G2/M phases of the cell cycle when treated with DMSO control (green), cisplatin 100µM (light blue), SHG-8 10µM (dark blue) and SHG-8 40µM (pink) conditions. **(D)** ROS assay was utilised to determine if SHG8 could induce ROS production to infer a mechanism of action to promote apoptosis. Fluorescence intensity was measured at 520/605nm over a 2-hour period at 15 min intervals (N=4). Data is presented as mean ± SD. Two-way ANOVA followed by Dunnett’s multiple comparison *post-hoc* test was conducted. *P<0.05, **P<0.01, ***P<0.001, **** P<0.0001 were considered as statistically significant.

SHG-8 was evaluated on whether it could induce apoptotic cell death to produce its cytotoxic effect (**Figure 3**). DAPI staining was performed to see cell fragmentation as a marker of apoptosis. Initially, SHG-8 induced apoptosis in a dose-dependent manner with nuclear fragmentation occurring at both 10μM and 40μM concentrations (**Figure 3A**). Morphological changes of the cancer cells were also apparent; with irregular structural changes indicating a consequential effect of nuclear fragmentation; shown with white arrows. Furthermore, dual staining with Annexin-V and PI has shown significant apoptosis induction in cisplatin 100μM, SHG-8 10μM and 40μM conditions in comparison to the DMSO control (**Figure 3B**). The cisplatin condition had a larger percentage of cells within early apoptosis at 13.88% (lower right quadrant) whilst both SHG-8 10μM and 40μM conditions had a larger percentage of cells exhibiting late apoptosis induction (upper right quadrant) at 15.74% and 17.75% respectively when compared to DMSO. All conditions have shown a minimal number of cells exhibiting necrotic like features (upper left quadrant). Moreover, cell cycle arrest analysis of SHG-8 treated SW480 cells have shown an increased presence in the G2/M phase with SHG-8 at 10μM (13.314%) and 40μM (16.168%) which demonstrated the greatest increase in comparison to the DMSO control condition (10.968%) (**Figure 3C**). Cisplatin shows the greatest number of cells present in the S phase (12.802%) of the cell cycle in comparison to SHG-8 treatments of 10μM and 40μM at 5.954% and 5.975% respectively. Further to this, there are minimal cell populations present in the sub-G0 phase in all treatment conditions. Additionally, a ROS assay was performed to investigate the role of ROS production on the likelihood of apoptosis induction (**Figure 3D**). From this, it can be inferred that ROS production seemingly exhibited no effect on cell death.

### 3.4 Molecular docking of Siglec-15 and expression analysis

**Figure 4:**
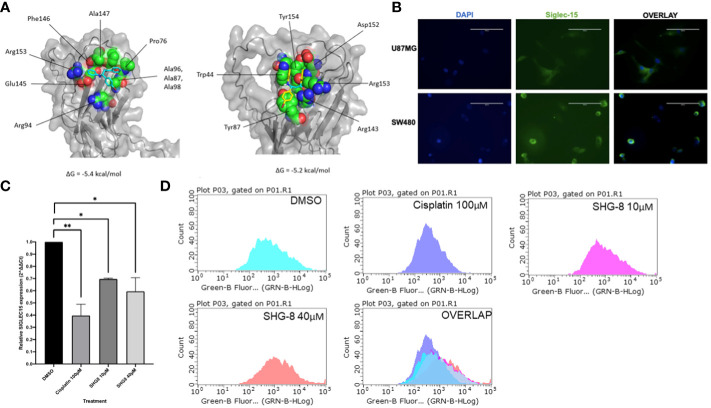
The effect of SHG8 treatment on *SIGLEC15* expression. **(A)** Docking study for SHG-8 binding on the Siglec-15 protein structure. **(B)** Positive Siglec-15 protein expression validated *in vitro* on SW480 CRC cancer cells in comparison to the U87MG Siglec-15 positive cell line (N=3). **(C)** *SIGLEC15* expression across various treated SHG-8 conditions 24h post- treatment via RT-qPCR methods (N=3). SHG-8 was shown to reduce *SIGLEC15* expression *in vitro*. One-way ANOVA statistical test followed by Dunnett’s multiple comparison *post-hoc* test, *P<0.05, **P<0.01. **(D)** Flow cytometry analysis of Siglec-15 protein expression of SW480 cells when treated with DMSO control (light blue), cisplatin 100µM (dark blue), SHG-8 10µM (pink) and SHG-8 40µM (red). There was no significant difference in the level of protein expression upon SHG-8 treatment exposure.

The top ranked binding site predicted by the COACH server included the amino acid residues of Siglec-15 at positions: 44, 70, 143,152,153,154,155 and 157 (**Supplementary Figure 1**) and the region selected as the target site for docking was predicted with very high confidence by AlphaFold. The highest predicted binding affinity between SHG-8 and Siglec-15 was -5.4 kcal/mol and the docking pose revealed that SHG-8 binds around a narrow cleft on the surface of the protein. The amino acid residues that form the cleft and are within 5.0 Å of SHG-8 include: Arg153, Glu145, Ala147, Phe146, Pro76, Ala97, Ala96, Ala98, Arg94 and Ala107 (**Figure 4A**). The binding affinity for mode two was -5.2 kcal/mol. The amino acid residues with atoms within 5.0 Å of SHG-8 mode 2 include: Trp44, Tyr87, Tyr154, Arg143, Asp152, Arg153. Later investigations looked at Siglec-15 expression *in vitro* which tested positive for expression with a highly expressed Siglec-15 positive cell line (U87MG) for comparison via fluorescence staining methods (**Figure 4B**). RT-qPCR methods were used to determine the expression of *SIGLEC15* when treated with SHG-8. RT-qPCR methods have shown that SHG-8 regulates *SIGLEC15* expression. We have identified a downregulation of *SIGLEC15* respectively at both SHG-8 10µM and 40µM treated conditions (*p<0.5, p<0.01, p<0.0001*) (**Figure 4C**). However, Siglec-15 protein expression via flow cytometry methods highlighted no significant difference in expression level when treated with SHG-8 at 10μM and 40μM conditions in comparison to the DMSO control (**Figure 4D**).

### 3.5 SHG-8 toxicity is reduced in colon epithelial cells and inhibits pro-inflammatory cytokine secretion in stimulated macrophages

**Figure 5:**
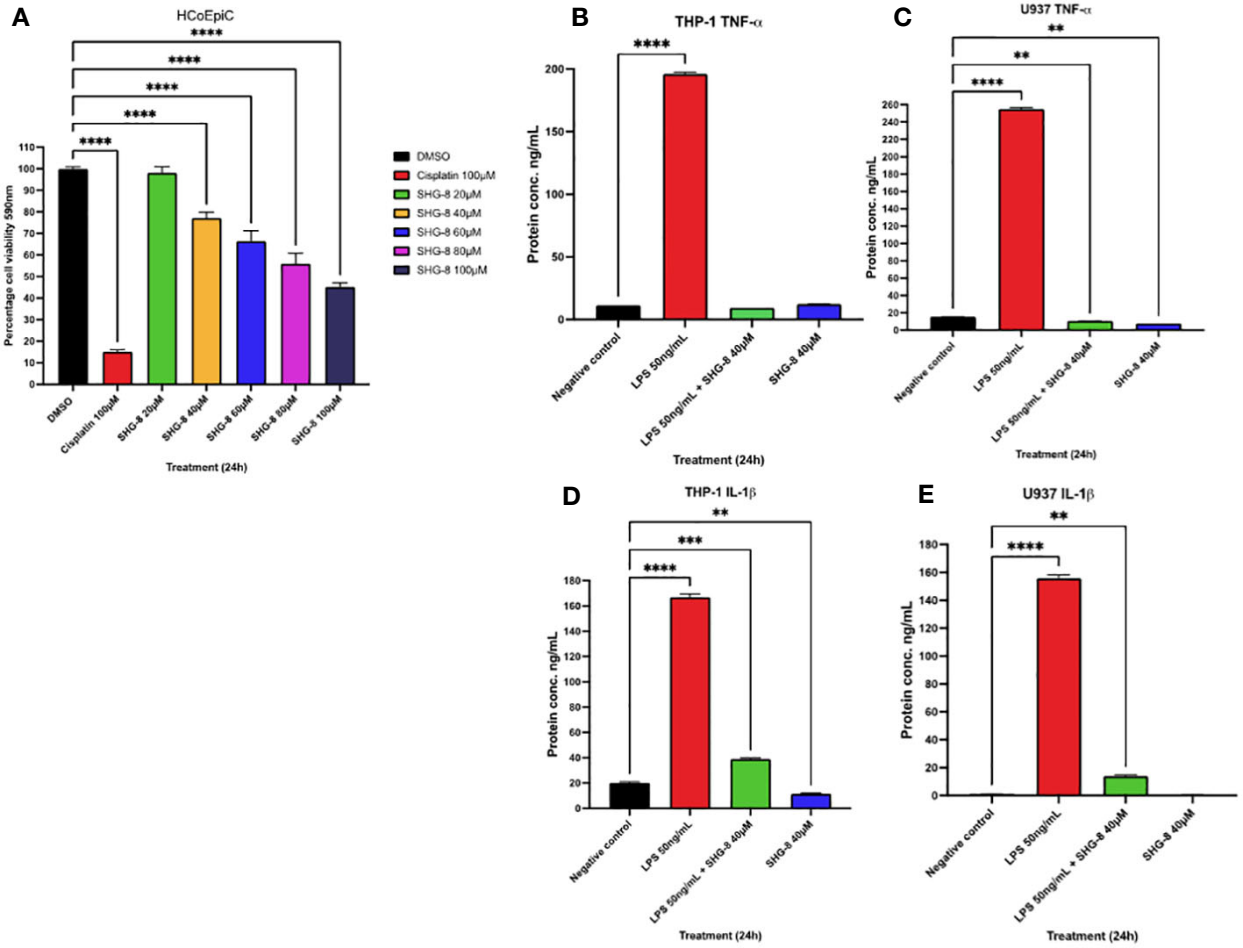
SHG-8 toxicity is reduced in colon epithelial cells and inhibits pro-inflammatory cytokine secretion in stimulated macrophages **(A)** MTT assay was conducted on normal colon epithelial HCoEPiC cells to determine the IC_50_ of SHG-8 24h post-treatment and to evaluate its cytotoxic effects *in vitro*. Data is presented as mean ± SEM. One-way ANOVA statistical test followed by Dunnett’s multiple comparison *post-hoc* test **** P<0.0001. **(B)** TNF-α cytokine secretion in LPS stimulated THP-1 differentiated macrophages post SHG-8 exposure. SHG-8 normalises TNF-α secretion following LPS stimulation. Data is presented as mean ± SD. One-way ANOVA statistical test followed by Dunnett’s multiple comparison *post-hoc* test **** P<0.0001. **(C)** TNF-α cytokine secretion in LPS stimulated U937 differentiated macrophages post SHG-8 exposure. SHG-8 normalises TNF-α secretion in stimulated macrophages and inhibited the presence of the pro-inflammatory cytokine at the 40μM only condition. Data is presented as mean ± SD. One-way ANOVA statistical test followed by Dunnett’s multiple comparison *post-hoc* test **** P<0.0001, ** 0.01. **(D)** IL-1β cytokine secretion in LPS stimulated THP-1 differentiated macrophages post SHG-8 exposure. SHG-8 reduced IL-1β secretion and inhibited the presence of the pro-inflammatory cytokine at 40μM only condition in comparison to the control group. Data is presented as mean ± SD. One-way ANOVA statistical test followed by Dunnett’s multiple comparison *post-hoc* test **** P<0.0001, *** 0.001, **0.01. **(E)** IL-1β cytokine secretion in LPS stimulated U937 differentiated macrophages post SHG-8 exposure. SHG-8 reduced IL-1β secretion following LPS stimulation. Data is presented as mean ± SD. One-way ANOVA statistical test followed by Dunnett’s multiple comparison *post-hoc* test **** P<0.0001, **0.01.

To determine if SHG-8 poses a cytotoxic effect on normal colon epithelial cells, an MTT assay was conducted using HCoEPiC cells at varying SHG-8 concentrations (**Figure 5A**). SHG-8 has shown no significant difference in cell viability at 20µM which was the determined IC_50_ value in SW480 cells. However, increasing SHG-8 concentrations affect cell viability ranging from 77.18% at 40µM to 45.26% at 100µM concentration in comparison to 32.97% at 40µM to 5.41% at 100µM at the same concentration range in SW480 cells. Moreover, the IC_50_ value for HCoEPiC treated cells was found around 90µM, which is significantly higher than SW480 cells highlighting lower toxicity. In addition to this, to determine the effect of pro-inflammatory cytokine secretion in immune cells in the presence of SHG-8, the cytokine levels of TNF-α and IL-1β were determined 24h post SHG-8 treatment and LPS (50ng/mL) stimulation (**Figures 5B-E**). LPS stimulation significantly enhanced pro-inflammatory cytokine secretion of TNF-α and IL-1β in both differentiated macrophage cell lines in comparison to the control group (p<0.0001). In addition to this, SHG-8 treatment at 40µM reduced the LPS-triggered pro-inflammatory cytokine release from differentiated macrophages in all variable conditions. Furthermore, upon exposure to SHG-8 only at 24h, there is a significant reduction in inflammatory cytokine secretion with lower TNF-α secreted in U937 (7.731ng/mL) and IL-1β in THP-1 cells (11.52ng/mL).

### 3.6 sRNA-seq revealed dysregulation of miRNAs by the treatment of SHG-8 on CRC cells

**Figure 6:**
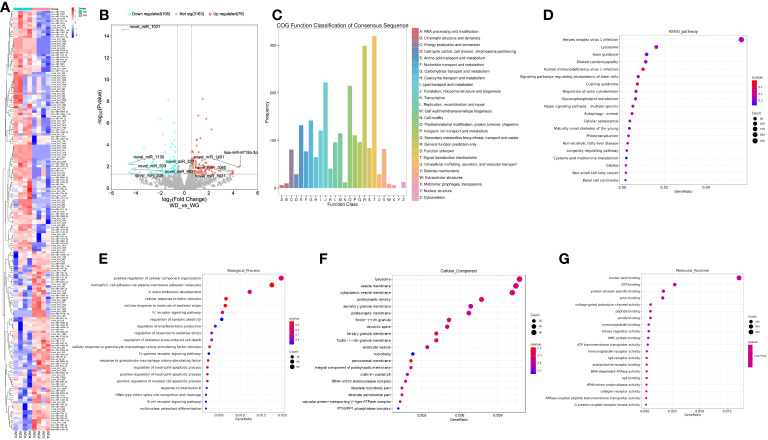
SHG-8 induces differential expression of miRNAs in CRC cells. **(A)** A constructed heat map illustrating differential miRNA expression in the SHG-8 treated and untreated group. **(B)** A volcano plot was constructed to illustrate the top 5 upregulated and downregulated miRNAs between the SHG-8 treated and untreated groups. **(C)** COG functional analysis was conducted to determine the differential expression of miRNAs and associated enriched pathways. **(D)** KEGG pathway analysis identifying enriched signalling pathways between SHG-8 treated and untreated groups. **(E)** GO classification determining enriched biological processes relating to differentially expressed miRNAs. **(F)** GO classification for enriched cellular components relating to differentially expressed miRNAs. **(G)** GO classification for molecular function relating to differentially expressed miRNAs.

SRNA-seq analysis of mRNA SHG-8 treated samples identified clear differential miRNA expression between SHG-8 treatment and control conditions via constructed heat map (**Figure 6A**). Analysis also identified 185 differentially expressed miRNAs via volcano plot (**Figure 6B**). Of those, 106 genes were identified as significantly downregulated, and 79 genes were significantly upregulated having shown a two-fold change in expression. The top 5 most significantly downregulated miRNAs were identified as novel miR-1031/1130/503/993 whilst the top 5 most significantly upregulated miRNAs were listed as novel miR-1401/1065/233/1431 and hsa-miR-6715b-3p. Differential expression also classified COG functions which were correlated largely with signal transduction mechanisms and functions related to translation, ribosomal structure, and biogenesis (**Figure 6C**). KEGG pathway analysis depicted that signalling pathways related to autophagy and senescence were all enriched (**Figure 6D**). GO classification identified several biological processes were elevated including regulation of cellular components, cellular response, and regulation of immune cell apoptotic processes (**Figure 6E**). Additionally, cellular components relating to lysosomes and vesicles were enriched (**Figure 6F**). Furthermore, nucleic acid binding displayed the most enriched molecular function followed by GTP binding and specific protein domain binding (**Figure 6G**).

### 3.6 RT-qPCR and *in silico* analysis of miR-6715b-3p and *PTTG1IP* in SW480 cells

**Figure 7:**
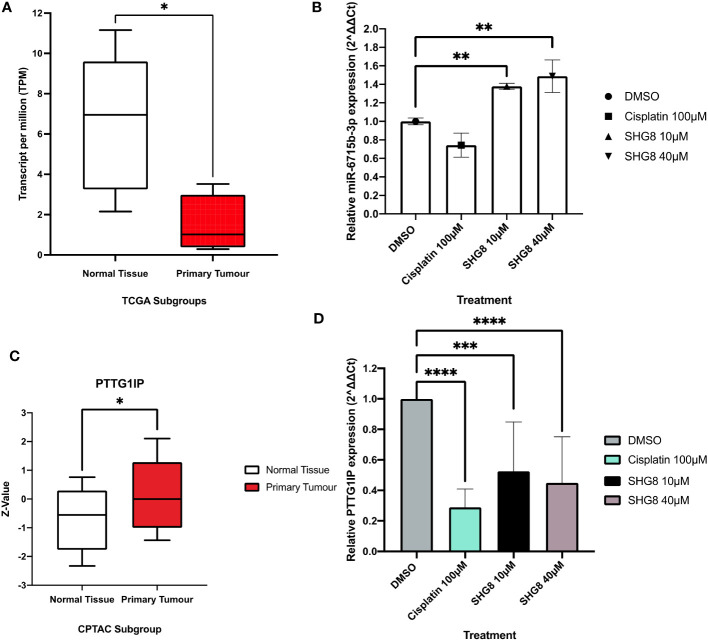
*In silico* analysis and RT-qPCR validation of sRNA-seq analysis. **(A)** TCGA genomic data for miR-6715b-3p expression in normal (clear) and CRC tumour groups (red) box plots. Unpaired student’s t-test *P<0.05. **(B)** Relative expression of miR-6715b-3p in SHG-8 treated and untreated conditions *in vitro*. One way ANOVA followed by Dunnett’s multiple comparison *post-hoc* test **P<0.01. (N=3). **(C)** TCGA proteomics data for PTTG1IP expression in normal (clear) and CRC tumour groups (red) box plots. Unpaired student’s t-test *P<0.05. **(D)** Relative expression of *PTTG1IP* in SHG-8 treated and untreated conditions *in vitro*. One way ANOVA followed by Dunnett’s multiple comparison *post-hoc* test ***P<0.001, ****P<0.0001. (N=3).

To investigate the expression profile of miR-6715b-3p in CRC, UALCAN TCGA genomic data was used to confirm sRNA-seq findings (**Figure 7**) and analysed its expression in the CRC tumour group compared to a normal subgroup (**Figure 7A**). The mean expression level of miR-6715b-3p in the tumour group was 0.655 while in the normal tissue subgroup, the mean expression level was 6.949, indicating a significant substantial dysregulation (*p<0.05*) of miR-6715b-3p in CRC. To confirm the validity of miR-6715b-3p expression in CRC outlined via *in silico* analysis, RT-qPCR analysis determined the relative expression of miR-6715b-3p *in vitro* between SHG-8 treated and untreated conditions (**Figure 7B**). The resulting data demonstrated a significant increase in the expression of miR-6715b-3p (*p<0.01*) following SHG-8 treatment at both 10µM and 40µM concentrations in comparison to the untreated control. Therefore, confirming the observed increase in miR-6715b-3p expression identified by high-throughput sequencing. Furthermore, sRNA-seq analysis identified that miR-6715b-3p is involved in regulating *PTTG1IP*, a possible oncogene in CRC. To investigate the differential expression of the PTTG1IP protein between normal and tumour subgroups, *in silico* analysis was performed using proteomics data (**Figure 7C**). *In silico* analysis revealed significant upregulation of PTTG1IP protein expression in the CRC tumour subgroup with a mean expression of 0.00 compared to their corresponding normal tissue subgroup with a mean expression of -0.554 (*p<0.05*). To assess the expression profile of *PTTG1IP* at the gene level in CRC cells, RT-qPCR analysis on SHG-8 treated and control subgroups was conducted to evaluate its relevance in CRC (**Figure 7D**). The RT-qPCR results revealed that *PTTG1IP* expression was significantly downregulated in both 10µM and 40µM SHG-8 treated conditions with a relative expression of 0.525 and 0.449 respectively compared to the control group (*p<0.01, p<0.001*). The mean relative expression of *PTTG1IP* in treated SW480 cells were 1.12-fold lower than in untreated cells, indicating an SHG8-treatment induced effect on *PTTG1IP* regulation in SW480 cells.

## 4 Discussion

To treat CRC cases, conventional front-line treatments would involve the usage of chemotherapy, surgery and radiotherapy or a combination of each to improve patient response (21). Despite several advances in CRC diagnosis, prognosis, and disease management and even development of new treatment strategies, ultimately it has fallen short (22). In last few decades, the targeted anticancer drugs including small molecules and macromolecules, have gained much attention due to their ability to specifically target cancer cells and spare normal cells and thus they possess high potency and low toxicity. In comparison to macromolecular targeted drugs, the small molecule targeted drugs have several advantages such as low cost, better pharmacokinetic properties, patient compliance, and easy storage and transportation and therefore considered favourable for anti-cancer drug development (23).

In recent years, small molecule targeted therapies have emerged as a promising approach for the treatment of various cancers including CRC preventing metastasis and cancer progression (24). Bearing in mind the significance of small molecules for CRC, we decided to explore the efficacy of the β-amino ketone (SHG-8) for CRC by targeting the Siglec-15/Sia axis.

The SHG-8 compound is a small molecule that contains three aromatic rings connected by a β-amino ketone. A halogen group is introduced to an aromatic ring to enhance lipophilicity and hydrophobicity. The efficient synthesis of β-amino ketones can be achieved through a one-step three-component Mannich reaction involving an amine, aldehyde, ketone, and a catalyst (25). Various catalytic systems have been explored, however, they suffer from drawbacks such as long reaction times, high temperatures, cost, low recyclability, and challenges in product separation and purification (26). Moreover, some catalysts pose environmental risks due to their corrosiveness and toxicity. In line with the United Nations Sustainable Goals (SDGs), a suitable and recyclable heterogenous catalyst for the Mannich reaction was developed. The heterogenous nano-catalyst SAFSNS, was synthesised and characterized by our reported procedure (15).

This study presents the development of an efficient, environmentally friendly catalytic process using a solid acid nano-catalyst (SAFSNS) for the one-pot condensation of aromatic ketones, aldehydes, and amines to yield the β-amino carbonyl compound (SHG-8). Further investigations are underway to explore the synthesis of a range of β-amino carbonyl compounds using the SAFSNS nano-catalyst.

It’s evident from the experimental results that the β-amino ketone compound SHG-8 has significant cytotoxicity *in vitro*. At an IC_50_ of approximately 20µM, SHG-8 has shown a higher treatment efficacy in comparison to current available treatments such as cisplatin. It has been reported that cisplatin displayed an IC_50_ of 23.61µM in CRC cells (27). Similar to what was observed in this study. This suggests the use of SHG-8 as a small molecule inhibitor as more effective at displaying anti-tumorigenic properties in comparison to cisplatin. Similarly, combination therapies were required to increase the efficacy of cisplatin drug treatment due to cancer cells developing resistance (28). In addition, wound healing assays demonstrated that cisplatin and the 40μM SHG-8 treated condition significantly reduced cellular migration across 24 hours. However, at the 48-hour time point, only cisplatin was able to achieve this effect. Moreover, all treated conditions displayed significant inhibition on cell proliferation as no colonies were formed. This also correlated similarly with other treatments including combination therapies and small molecule inhibitors inhibiting the proliferation of CRC cells *in vitro* (29).

The nuclear fragmentation assay inferred the occurrence of apoptotic cell death at increasing SHG-8 concentrations. This observation correlated with several other compounds including conventional and non-conventional treatment methods such as cisplatin which are also capable of modulating apoptosis in multiple cancer types (30). These images corresponded with cellular features commonly associated with apoptosis including nucleus fragmentation, irregular morphology, and cell shrinkage via caspase functional activity cleaving peptide bonds of nuclear proteins (31). In contrast, induced cell cycle arrest at the G2/M phase post-treatment observed via PI staining suggests prolonged generation of double stranded breaks within the DNA helical structure, hence the possibility of apoptosis induction (32). However, the rate of apoptosis induced by SHG-8 treatment was rather low, thus suggesting that other types of cell death pathways may be triggered by SHG-8.

The transition from G2 to M phase is highly regulated via several proteins including p53. Cellular exposure to SHG-8 might enhance the activity of p53 prompting p53-dependant G2 cell cycle arrest thereby inhibiting cancer proliferation (33). It could further propose specific mechanistic action of SHG-8 to inhibit tumour proliferation. Furthermore, SHG-8 exhibits similar apoptotic induction to other small molecule inhibitors including DMOCPTL, MLN4924 and RGX-202 used in several cancer types including triple negative breast cancer (TNBC), lung cancer and CRC (34–36). Additionally, there are previous reports stating that cisplatin significantly enhanced the presence of cells within the S phase of the cell cycle, this is in agreement with our findings (37). Another possible mechanistic explanation may involve ROS production and could be the underlying cause for which apoptosis occurs. Overstimulated ROS production within a tumour microenvironment could cause severe damage to various proteins and metabolites inducing the activation of apoptosis and related signalling pathways (38). However, this is not the case of SHG-8 as ROS production remained consistent throughout with only the positive control showing significant ROS generation. This suggested that apoptosis induction is modulated via a similar yet distinct signalling pathway, although the exact mechanism of activation remains unclear up to this point (39).

There is also growing evidence associating *SIGLEC15* upregulation with the development of cancer and its role in the progression of various types of tumours comprising of head and neck squamous cell carcinomas (HNSCC), liver hepatocellular carcinomas (LIHC), lung adenocarcinoma (LUAD), prostate adenocarcinoma (PRAD), rectum adenocarcinoma (READ), thyroid carcinoma (THCA) and many others including colorectal adenocarcinomas (COAD) tumours (7, 8, 40). Simultaneously, immune checkpoint proteins including Siglec-15 and PD-L1 have specified key roles in the proliferation of cancer cells and are able to modulate cancer progression (41, 42). In particular, Siglec-15 is also expressed on mutually exclusive populations of cancer cells with respect to PD-L1 (43). Therefore, this could pave the way for combination therapies with PD-L1 and Siglec-15 antagonists. Thus, in this study, elucidating candidate miRNAs as therapeutic targets for CRC progression could provide insights to treating Siglec-15 positive tumours and PD-L1 negative tumour patients.

Notably, it is understood that Siglec-15 functional activity requires canonical glycan binding. It has been reported that interactions with essential arginine amino acid residues were necessary for the formation of salt bridges with sialic acid carboxylates (44). Our docking studies aimed to elucidate potential binding sites of the Siglec-15 protein structure. The docking study located amino acid residues within 5.0 Å of SHG-8 including ARG143, an amino acid residue that has been previously reported for glycan ligand binding (45). Thereby suggesting SHG-8’s role as a small molecule inhibitor.

To determine the role of SHG-8 on CRC, Siglec-15 was shown to have positive expression *in vitro*. With SHG-8 treatment, RT-qPCR methods indicated *SIGLEC15* expression was significantly reduced. Several mechanisms maybe involved in the regulation of *SIGLEC15* expression. A previous report identified *hsa-miR-582-5p/TUG1* axis to be involved in pancreatic adenocarcinomas (PAAD) (46). Similarly, the *LINC02432/hsa-miR-98–5p/HK2* axis also correlated with *SIGLEC15* regulation in hepatocellular carcinomas (HCC) (47). Therefore, it may be possible that *SIGLEC15* is regulated through a similar mechanism. However, we have found no significant difference in Siglec-15 protein expression upon SHG-8 treatment. It may be possible that Siglec-15 binding and regulation at the gene level could in fact regulate apoptosis induction and subsequent pathways resulting in cancer cell death. It was previously reported that Siglec-15 knockdown could reduce STAT3 signalling thus inhibiting cellular proliferation and inducing apoptosis in osteosarcomas (48).

The effect of SHG-8 on HCoEPiC cells has demonstrated that the IC_50_ value at 90μM shares significantly lower potency in epithelial cells in comparison to the IC_50_ value at 20μM in SW480 cells. Similarly, a previous study highlighted Actein, a triterpene glycoside, significantly inhibited SW480 and HT-29 cells *in vitro* whilst exhibiting reduced anti-proliferation effects in HCoEPiC cells (49). Furthermore, MOG13 a selective inhibitor for CRC treatment, witnessed a concentration-dependant reduction in cellular viability when exposed to drug treatment (50). This could suggest that significantly higher concentrations from 40μM onwards could sensitise HCoEPiC cells towards SHG-8 treatment. Although, there was cell death at higher SHG-8 concentrations, the toxicity that was observed emphasises the effectiveness of SHG-8 as a treatment alternative and suggests reduced adverse effects in comparison to cisplatin treatment.

Chronic inflammation can play a crucial role in cancer development and progression (51). Secretion of pro-inflammatory cytokines including TNF-α and IL-1β can intrinsically contribute to the formation of the TME and systemic immunosuppression could be stimulated by cell populations including TAMs (52). Consequently, resulting in chronic inflammation and cancer progression. SHG-8 exposure has indicated a suppressive effect on the secretion of both TNF-α and IL-1β in the presence of LPS stimulated macrophages highlighting a possibility in preventing the occurrence of inflammation and the TME. In addition to this, reduced inflammatory cytokine secretion could also correlate with negligible ROS production as was seen in the ROS assay for SW480 cells.

The constructed heat map identified several clusters of miRNAs that were differentially expressed between the SHG-8 treated and untreated groups. Mir-1303 is one such differentially expressed miRNA which showed downregulated expression in the treatment group. This miRNA has typically shown upregulated expression and was capable of inducing tumour cell proliferation and invasion in various cancer types including CRC (53). Likewise, miR-940 has shown differential expression displaying downregulated expression in the treatment group. MiR-940 upregulation has consistently been shown to contribute to tumour progression in cancer types such as PAAD and cervical carcinomas (CC) (54). Moreover, miR-543 was shown to be downregulated in several tumour types including CRC by exhibiting a tumour suppressive role and inhibiting tumorigenesis (55). In the treatment group, miR-543 has shown increased expression supporting the conclusion in its role as a tumour suppressor. In addition to this, there have also been several reports of anti-tumorigenic drug compounds capable of affecting miRNA expression (56–58). Therefore, it is highly likely that SHG-8 could is involved in modulating miRNA expression profiles in order to inhibit cancer progression.

The differential expression for many miRNAs has shown that SHG-8 is compatible as a small molecule inhibitor and is capable of inducing changes to miRNA expression to inhibit cancer proliferation. To answer the question of whether the downregulation of *SIGLEC15* is mediated by miRNAs, sRNA-seq analysis was performed between SHG-8 treated and untreated conditions. Several miRNAs were shown to be differentially expressed with the SHG-8 treatment; however, we did not find miRNAs that significantly affect *SIGLEC15* expression with the treatment. The downregulation of Siglec-15 could be an effect of DNA methylation and/or by an effect of transcriptional regulation. Nevertheless, we found miR-6715b-3p to be the most upregulated miRNA. With minimal evidence in the literature for the novel miRNAs, miR-6715b-3p has been previously identified as integral in the modulation of autophagy through SESN1 targeting in Huntington’s disease and was also found to be downregulated in prostate adenocarcinomas (59, 60). In this study, *in silico* analysis and RT-qPCR methods confirmed miR-6715b-3p expression *in vitro* in SHG-8 treated conditions. It is possible that miR-6715b-3p acts as a tumour suppressor miRNA and could regulate oncogenes in regard to CRC progression. It has been reported that miR-6715b-3p could work in conjunction with other miRNAs to display superior anti-proliferative activity such as miR-34a (61). Supporting the likelihood that miR-6715b-3p could possess an important role in regulating key gene targets associated with CRC progression and improve on current therapeutics.

sRNA analysis has shown gene targets to be involved in multiple pathways and biological/molecular functions. KEGG pathway analysis detailed enriched signalling pathways related to autophagy and senescence when subjected to SHG-8 treatment. It is possible that miRNAs can regulate these pathways to inhibit tumorigenesis. Several miRNAs including miR-145 have been reported to promote autophagy via various signalling pathways (62–64). Likewise, several miRNAs including miR-15/17/19/21/24/29/34/101 have shown signs of modulating senescence in cancer development (65). MiRNA’s have also shown involvement in tumorigenic signalling regulation as well.

Several biological processes that were enriched included the regulation of immune cell apoptosis and cellular response. It is possible that miRNAs can regulate apoptotic processes in cancer. Mir-448 and miR-148a-3p were both reported to regulate immune cell apoptosis which inhibited cancer progression (66, 67).

Based on differentially expressed miRNAs induced via SHG-8 treatment; miR-6715b-3p was predicted to interact and significantly regulate *PTTG1IP*, a possible oncogene in CRC. Proteomics data outlined higher levels of PTTG1IP expression in *COAD* tumours with significant downregulation in the SHG-8 treated conditions confirming sequencing analysis. The expression of *PTTG1IP* has been reported in other malignant cancers including, CRC breast and thyroid cancers supporting its role as an oncogene (68–70). However, *PTTG1IP* has also shown low expression in malignancies which correlated with poor survival (71). It was also highlighted that the overexpression of *PTTG1IP* in malignant tumours are the main driving force for tumour progression whilst genetic mutations are likely to establish minimal effects on *PTTG1IP* function (72). Although, there are conflicting reports for the expression of *PTTG1IP* in malignant tumours, further assessment of the mechanistic action of the miR-6715b-3p/*PTTG1IP* axis in correlation with the Siglec-15/Sia axis could shed some light on CRC progression and maybe a promising approach for treatment.

Subsequent studies to decipher further mechanistic action of SHG-8 on cancer survival could elaborate on key intrinsic pathways particularly regarding the induction of apoptosis. Caspases and their cleaved counterparts including cleaved caspase-3 and cleaved caspase-9 maybe involved in the induction of apoptosis as a possible method for cancer death to exert SHG-8’s therapeutic potential (73). Furthermore, the expression of *SIGLEC15* at the gene level demonstrated reduced expression from SHG-8 treatment, knockdown studies may provide further mechanistic insights in SHG-8’s therapeutic potential. Similarly, we hypothesise that the role of SHG-8 as a Siglec-15 antagonist will have no effect on the expression of PD-L1. However, due to the mutual exclusivity in the expression of both inhibitory checkpoints, SHG-8 targeting of Siglec-15 in combination with PD-L1 antagonists may share synergistic effects. Further experimental analysis could underline and discern their role in cancer progression. Moreover, it may offer a more complete and robust approach in CRC treatment.

## 5 Conclusion

To conclude the small molecule SHG-8 has shown significant anti-tumour properties against SW480 cancer cells. Although indications do identify that SHG-8 does induce late apoptosis at higher concentrations with elevated cell cycle arrest at the G2/M phase of the cell cycle, further mechanistic studies are needed to gain insights as to how this phenomenon is generated. The molecule SHG-8 binding to ARG143 confirms its role as a novel small molecule inhibitor in Siglec-15 positive tumours. Moreover, it outlines the tumorigenic role of the Siglec-15/Sia and miR-6715b-3p/*PTTG1IP* axes in the progression of CRC. It is worth noting that Siglec-15 expression is exclusively expressed on distinct subpopulations of cancer cells with respect to PDL-1. Therefore, our study indicates the possibility of combination therapies utilising both PDL-1 and Siglec-15 antagonists to induce a successful patient response and prevent tumour recurrence. These findings offer a promising avenue for future investigations for CRC treatment. Notably, SHG-8 is the first known inhibitor to target Siglec-15, with current clinical trials so far has only focused on the development of blocking antibodies to inhibit the Siglec-15/Sia axis. SHG-8 could be a gamechanger in the way CRC and other Siglec-15 positive cancers will be treated in the future.

## Data availability statement

The data presented in the study are deposited in the Mendeley repository, accession number doi: 10.17632/r2xdf868k9.1; release date 3 Aug 2023.

## Ethics statement

Ethical approval was not required for the studies on humans in accordance with the local legislation and institutional requirements because only commercially available established cell lines were used.

## Author contributions

MA: data curation, methodology, writing – original draft, writing – review & editing. MB: conceptualization, supervision, writing – review & editing. HP: data curation, methodology, writing – original draft, writing – review & editing. IA: data curation, funding acquisition, methodology, writing – original draft, writing – review & editing. S: conceptualization, data curation, methodology, writing – original draft, writing – review & editing. SS: conceptualization, project administration, supervision, writing – review & editing.

## Glossary

**Table:**

SHG8	3-(4-bromophenyl)-1-phenyl-3-(phenylamino)propan-1-one
CC	cervical carcinomas
COAD	Colorectal adenocarcinomas
CRC	Colorectal cancer
ESI	Electrospray ionization
ESI-MS	Electrospray ionization Mass spectra
FACS	fluorescence-activated cell sorting
GO	Gene Ontology
GSEA	Gene Set Enrichment Analysis
HCC	Hepatocellular carcinomas
HNSCC	Head and Neck Squamous Cell Carcinomas
HRMS	High Resolution Mass Spectra
IF	Immunofluorescence
KEGG	Kyoto Encyclopaedia of Genes and Genomes
LIHC	Liver Hepatocellular Carcinomas
LUAD	Lung Adenocarcinoma
MiRNAs	MicroRNAs
MSI-H	High Microsatellite Instability
NSCLC	Non-Small Cell Lung Cancer
OV	Ovarian Cancer
PAAD	Pancreatic Adenocarcinomas
PRAD	Prostate Adenocarcinoma
PI	Propidium Iodide
READ	Rectum Adenocarcinoma
ROS	Reactive Oxygen Species
RT	Room Temperature
RT-qPCR	Real Time- Quantitative Polymerase Chain Reaction
SAFSNS	Sulfonic Acid-Functionalized Silica Nanospheres
SCC	Lung Squamous Cell Carcinoma
SDG’s	United Nations Sustainable Goals
Siglec-15	Sialic acid-binding immunoglobulin-type of lectin 15
THCA	Thyroid Carcinoma
TLC	Thin-layer Chromatography
UTR	Untranslated Region.
