# Supplementary figures and images for: Novel Siglec-15-Sia axis inhibitor leads to colorectal cancer cell death by targeting miR-6715b-3p and oncogenes
Source: Front Immunol. 2023 Oct 6;14:1254911. doi: 10.3389/fimmu.2023.1254911 (PMC10587484; doi:10.3389/fimmu.2023.1254911)

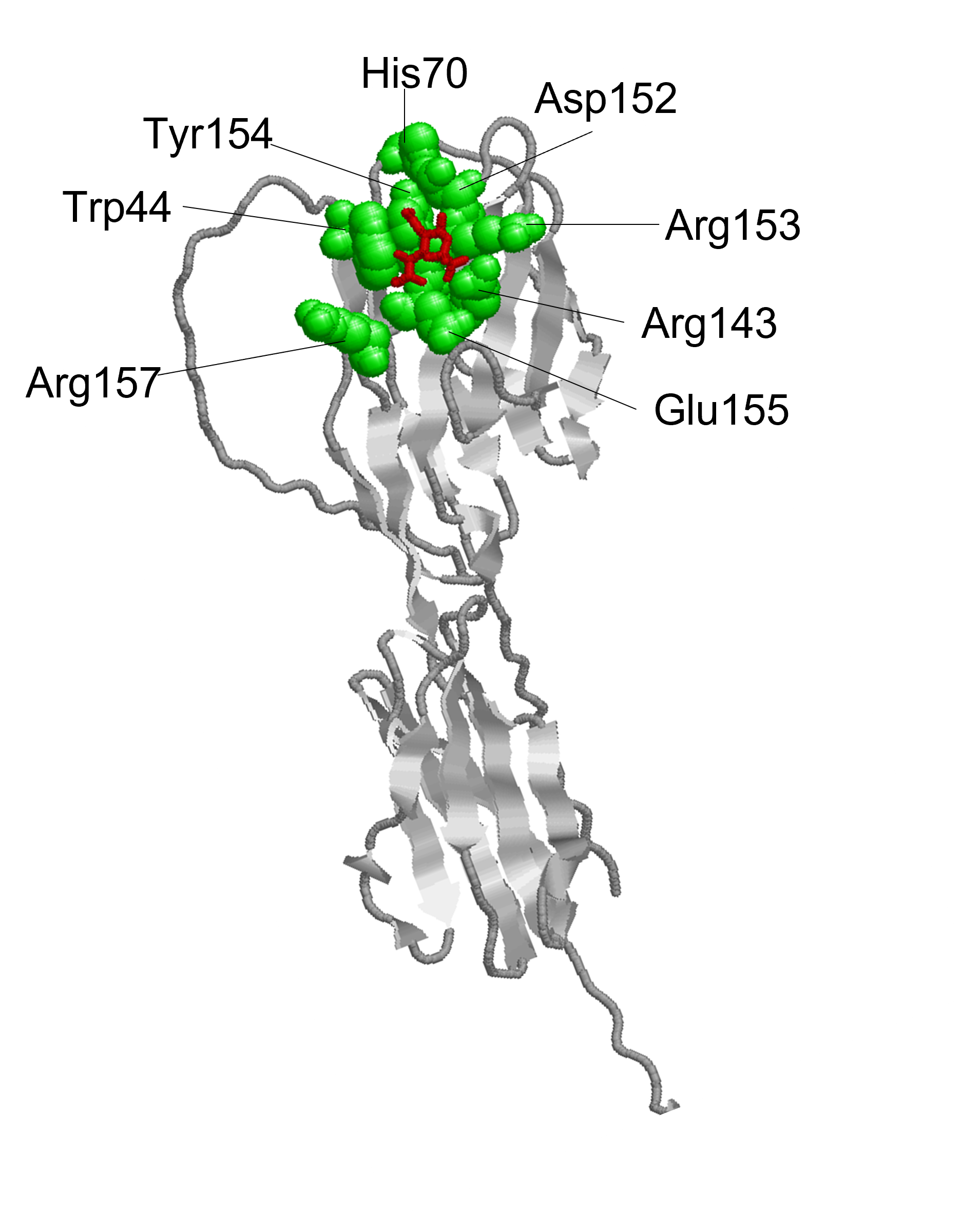

Supplement: Supplementary file 1 [file Image_1.tif]
